# Subthreshold Laser Titration Database in a Population with Central Serous Chorioretinopathy and Dome-Shaped Macula

**DOI:** 10.3390/jcm14030953

**Published:** 2025-02-02

**Authors:** Jacobo Emilio Enríquez-Fuentes, Alicia Valverde-Megías, Antonio Domingo Alarcón-García, Carlos Oribio-Quinto, Jay Chhablani, José Ignacio Fernández-Vigo

**Affiliations:** 1 Department of Ophthalmology, Hospital Clínico San Carlos, 28040 Madrid, Spain; jacef2020@gmail.com (J.E.E.-F.); valverdmegias@hotmail.com (A.V.-M.); antoniodomingo.alarcon96@gmail.com (A.D.A.-G.); carlosoribio@gmail.com (C.O.-Q.); 2 Department of Ophthalmology, School of Medicine, University of Pittsburgh, Pittsburgh, PA 15213, USA; jay.chhablani@gmail.com; 3 Centro Internacional de Oftalmología Avanzada, 28010 Madrid, Spain; 4 Department of Ophthalmology, School of Medicine, Complutense University of Madrid, 28040 Madrid, Spain

**Keywords:** subthreshold laser, micropulse laser, titration, central serous chorioretinopathy, dome-shaped macula

## Abstract

**Objectives:** This study aimed to describe the laser titration needed to create a safe and effective subthreshold laser (STL) for use in patients with chronic central serous chorioretinopathy (CSCR) and dome-shaped macula (DSM) and to investigate the possible factors influencing titration. **Methods**: This was a prospective single-center study that recruited 92 eyes of 87 patients that presented with CSCR (84.8%) and DSM (15.2%) for a 577 nm STL treatment for persistent subretinal fluid. Age, sex, and the spherical equivalent (SE) were collected. Titration was performed by applying various impacts, beginning at 800 mW and increasing in 100 mW steps. The photocoagulation threshold (PT) was assessed as the minimum power at which faint whitening was observed in fundus retinography. **Results**: The mean age was 53.5 ± 10.3 years and 78.3% of patients were male. The mean SE was 0.3 ± 3.0 D (range −9.5–+11.0). The overall mean power to reach PT was 1102.7 ± 183.0 mW (range 800–1600). In the multivariate analysis, age and SE were associated with PT (*p* = 0.022 and 0.011, respectively). On the contrary, no association was observed between PT and sex (*p* = 0.924), macular disease (CSCR vs. DSM, *p* = 0.416), or central macular thickness (CMT) (*p* = 0.667). **Conclusions**: This study highlights the broad power range required for effective subthreshold laser (STL) titration and emphasizes the need for individualized treatment parameters to optimize outcomes. No significant differences in titration power were found regarding macular disease type, CMT, or sex. However, a mild correlation between PT, SE, and age was observed.

## 1. Introduction

Subthreshold laser therapy (STL), or a micropulse laser (MP), has been utilized in the field of retinal treatment for more than twenty years [1]. This form of laser photocoagulation is unique because it does not produce visible clinical or histological retinal damage [2,3]. Instead, STL provides therapeutic benefits to the retinal pigment epithelium (RPE) by inducing the production of heat-shock proteins, which protect cells from stress by inhibiting the apoptotic and inflammatory pathways that lead to cellular damage [4]. Using a low-duty cycle prevents the continuous accumulation of thermal energy, allowing heat dissipation and reducing collateral damage to the neurosensory retina. This avoids coagulative necrosis and preserves retinal architecture. It is essential to distinguish between STL, which does not achieve photocoagulation, and subvisible laser therapy, which leaves no detectable trace [5].

STL has been increasingly adopted for promoting the resorption of subretinal fluid (SRF) in various macular conditions, particularly in central serous chorioretinopathy (CSCR) and dome-shaped macula (DSM) [6,7,8,9,10]. CSCR is a prevalent retinal disorder among middle-aged individuals. Although many cases resolve spontaneously, chronic CSCR can result in irreversible visual acuity (VA) loss due to persistent subretinal fluid and subsequent photoreceptor damage. While photodynamic therapy (PDT) has been considered the gold-standard treatment [11,12,13,14], the global shortage of verteporfin since May 2020 [15] has limited its availability, increasing interest in alternative treatment strategies such as STL [13,14].

DSM, on the other hand, is a structural abnormality observed in highly myopic patients. It is characterized by an inward bulging of the macula due to localized scleral thickening [16,17]. This condition is often associated with serous retinal detachment, and STL has shown promising results in reducing SRF and stabilizing vision in DSM patients.

Despite its growing application, one of the significant challenges of STL is the lack of a standardized protocol. Different laser settings have been proposed, but considerable variability exists in study parameters, including energy levels and titration strategies [1,6,7,18,19,20,21]. The first reported study using titrated yellow 577 nm STL for CSCR dates back to 2015 and involved only 15 eyes [22]. Since then, no clear consensus has been reached on the optimal parameters to ensure efficacy and safety.

A key point of controversy in STL treatment lies in the debate between personalized titration versus fixed-parameter approaches. One approach advocates for titrating the laser power individually before initiating treatment, tailoring the energy settings to each patient’s retinal characteristics [23]. Conversely, another approach supports using predefined fixed parameters to minimize the potential risks associated with titration variability [24]. Even among proponents of titration, there is no agreement on the ideal energy level: some recommend using one-third of the photocoagulation threshold (PT) power [25]. In contrast, others suggest using half [23], leading to discrepancies in clinical practice.

The main concern with STL treatment is the risk of over- or undertreatment. Excessive energy delivery can lead to unintended retinal damage, whereas insufficient power may render the treatment ineffective. Our research group previously described severe hyperplasia of the RPE (HRPE) in seven CSCR patients following STL, which resulted in significant VA impairment, likely due to overtreatment [26]. On the other hand, undertreatment has been widely recognized as the most frequent mistake among STL beginners, as applying insufficient energy may fail to achieve the necessary photothermal stimulation, leading to wasted treatment efforts and suboptimal outcomes in clinical trials.

**Figure 1:**
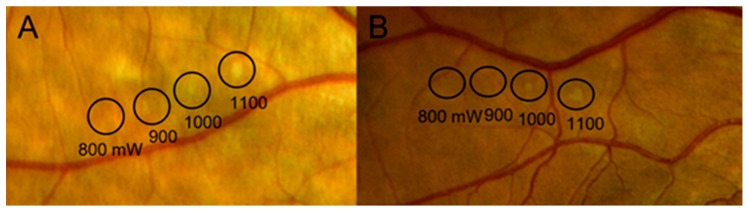
Retinal whitening due to the performance of the titrate used to achieve the photocoagulation threshold (PT). The power required to reach the PT was considered to be 1000 mW in both (**A**) and (**B**).

Given these challenges, there is a growing preference for a personalized STL approach, where titration is tailored to each individual patient (Figure 1). However, there is a critical gap in our knowledge regarding the reference values for PT required for appropriate titration, as well as the factors that may influence these thresholds.

The purpose of the present study is to describe the power needed to achieve the PT through STL in patients with CSCR and DSM to investigate its potential associations with ocular and demographic features.

## 2. Materials and Methods

### 2.1. Study Design and Sample

A cross-sectional study was conducted that included 92 eyes of 87 patients who received STL treatment for persistent SRF due to CSCR or DSM from December 2023 to June 2024 at the Retina Unit of the Department of Ophthalmology of the Hospital Clínico San Carlos (Madrid, Spain). The study protocol adhered to the principles of the Declaration of Helsinki. Informed consent was obtained from the patients recruited for this study.

### 2.2. Patients

The study’s inclusion criteria were a diagnosis of CSCR or DSM with at least 4 months of SRF persistence and being Caucasian. The diagnosis was confirmed based on established criteria for CSCR and DSM [27,28,29], and other possible causes of SRF were ruled out. Cases presenting a bulge of the RPE, choroid, and sclera inward that were more than 50 µm in height were classified as DSM. The study population was composed exclusively of Caucasian patients due to the demographic characteristics of the hospital’s patient base. This choice aimed to reduce confounding factors related to ethnic variations in retinal pigmentation, choroidal thickness, and laser absorption, which could influence titration power.

The exclusion criteria included active treatment with corticosteroids, other pathologies, previous treatments with anti-VEGF or PDT in the preceding 6 months, poor image quality, media opacities, or an incomplete examination.

Patients completed a pre-treatment visit where VA was measured using the Early Treatment Diabetic Retinopathy Study (ETDRS) chart. Macular optical coherence tomography (OCT) and autofluorescence (AF) were also performed. In cases where choroidal neovascularization (CNV) needed to be ruled out, OCT angiography (OCTA) was performed. The spherical equivalent (SE) was also measured using autorefraction.

### 2.3. Subthreshold Laser Therapy

Patients who met the inclusion criteria received navigated STL with a wavelength of 577 nm (Navilas 577s; OD-OS GmbH, Teltow, Germany). The laser treatment was performed by two experienced ophthalmologists (JIFV and AVM). Titration was planned using a true-color retinography performed with the device focused on areas of healthy RPE without atrophy, aided by an AF image, and was performed before treatment in all patients. The procedure began with a spot size of 100 µm, a duty cycle of 5%, a pulse duration of 200 ms, and an initial power of 800 mW. The titration process was designed to reach the photocoagulation threshold (PT) while minimizing interobserver variability. The initial starting point of 800 mW was based on previous studies and a preliminary pilot analysis conducted in our unit, where it was observed that only a minority of eyes reached the PT below this level. Incremental adjustments of 100 mW were chosen to allow precise control over the titration process while ensuring a balance between safety and efficacy. Each titration step was followed by a two-minute waiting period to assess for subtle retinal whitening, ensuring an accurate PT determination. Therefore, the PT was considered the lowest power necessary to achieve this faint whitening. The laser treatment was then applied to the area where SRF was observed using 33% of the energy determined by the PT; the pulse duration was 200 ms, with a duty cycle of 5% and a spot size of 150 µm.

### 2.4. Outcomes

Age, sex, the laterality of the treated eye, SE, central macular thickness (CMT), and the macular pathology of the patient were recorded. The overall power required to achieve the PT was obtained. Subsequently, the power needed to reach the PT was analyzed based on the aforementioned parameters. Patients were classified according to their SE: those between ±1.0 diopters (D) were considered emmetropes, with hyperopic patients having more than +1.0 D and myopic subjects having less than −1.0 D. The CMT was measured in micrometers from the internal limiting membrane (ILM) to the RPE in the OCT. The patients’ response to the treatment at 2 months was classified as follows: A total response (TR) if the SRF resolved after applying the STL and partial response (PR) if there was a decrease in SRF of at least 30 µm (a cut-off point considered to exclude variations due to different SRF measurements in the OCT), but without achieving TR. The remaining cases were considered no response (NR). The global response (GR) was considered the sum of TR and PR.

### 2.5. Statistical Analysis

The Statistical Package for Social Sciences (SPSS, v21.0; SPSS Inc., Chicago, IL, USA) was used for statistical analysis. Frequency and percentage statistics or the mean and standard deviation were calculated depending on whether the variables were qualitative or quantitative. The correlation between quantitative variables was assessed using Pearson correlations, and hypothesis contrast tests were performed to assess differences between different subgroups (Student’s *t*-test for independent data if the subgroups had fewer than 30 individuals, Mann–Whitney U if there were 2 subgroups, or Kruskal–Wallis if there were 3 subgroups). A multivariate stepwise linear regression analysis was conducted to identify the main factors influencing the power needed to reach the PT. A *p*-value of <0.05 was considered statistically significant.

## 3. Results

A total of 92 eyes from 87 patients were recruited. The mean age of the patients was 53.5 ± 10.3 years. The sample comprised 72 males (78.3%) and 20 females (21.7%). The treated eyes included 47 right eyes (OD) (51.1%) and 45 left eyes (OS) (48.9%). Of the treated eyes, 84.8% had CSCR and 15.2% had DSM. The mean CMT was 303.68 ± 101.33 µm (111–606). The mean SE was 0.3 ± 3.0 D, with 21.7% myopic, 42.4% emmetropic, and 35.9% hyperopic. Regarding the response to treatment, 16.3% showed a TR, 24.5% had a PR, and 40.8% achieved a GR, with 59.2% of the eyes showing NR.

### 3.1. Overall Power Needed to Achieve the PT According to Different Factors

**Figure 2:**
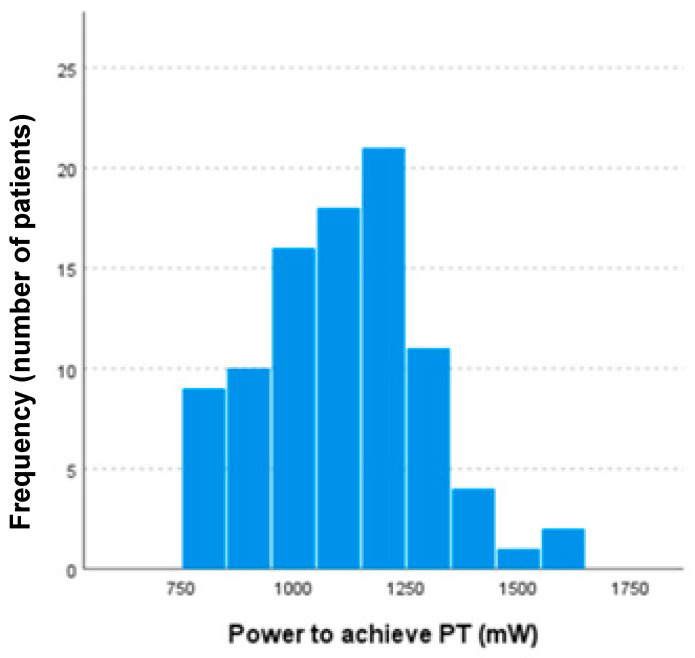
Frequency distribution of global power needed to achieve the photocoagulation threshold (PT).

The mean power required to achieve the PT was 1102.7 ± 183.0 mW, ranging from 800 (the protocol’s initial treatment setting) to 1600 mW (Figure 2). No differences in PT were observed according to gender, with the mean for men being 1102.8 ± 181.3 mW and that for women being 1102.5 ± 193.6 mW (*p* = 0.924). Similarly, no difference between OD and OS was found (1075.5 ± 174.4 mW for OD and 1131.1 ± 189.3 mW for OS, respectively (*p* = 0.146)). A weak correlation was found between SE and PT (*r* = −0.239, *p* = 0.022), while no correlation was observed between age and PT (*p* = 0.124) or CMT (*p* = 0.667).

**Figure 3:**
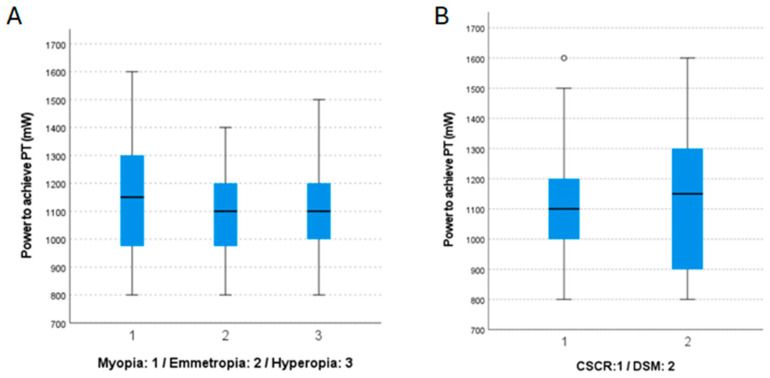
(**A**): Distribution of the power required to achieve the photocoagulation threshold (PT) between the myopia (1), emmetropia (2), and hyperopia (3) subgroups. (**B**): Distribution of the power required to achieve the PT between the chronic central serous chorioretinopathy (CSCR) (1) and dome-shaped macula (DSM) (2) subgroups.

There were also no differences in the PT among subgroups based on SE, with the mean being 1155.0 ± 234.5 mW for myopic patients, 1067.9 ± 157.1 mW for emmetropic patients, and 1112.1 ± 173.2 mW for hyperopic patients (*p* = 0.304) (Figure 3A). In terms of macular condition, no differences in PT were observed (1094.2 ± 172.6 mW for patients with CSCR and 1150.0 ± 234.5 mW for DSM; *p* = 0.416) (Figure 3B).

The observed wide range of power required to reach PT (800–1600 mW) highlights the challenge of using fixed-parameter approaches in STL treatment. Only 21.7% of eyes in our study had a PT of 1200 mW, a level commonly assumed in fixed regimens. This means that 58.7% of eyes would have been overtreated and 19.6% undertreated if fixed parameters were used. These findings support the necessity of individualized titration protocols to optimize treatment efficacy while minimizing risks. Additionally, the correlations observed between PT, age (*p* = 0.022), and spherical equivalent (*p* = 0.011) suggest that anatomical and physiological differences play a role in determining the required laser power. Our results align with previous studies reporting variability in STL responses but provide new insights into potential predictors of titration thresholds.

### 3.2. Multivariate Regression Analysis

In the multivariate regression analysis, refractive error and age remained determinants of the power needed to achieve the PT (*p* = 0.011 and *p* = 0.022, respectively) (Table 1). However, no association was found between PT and gender (*p* = 0.469), macular condition (*p* = 0.836), or CMT (*p* = 0.992).

## 4. Discussion

Determining the optimal STL laser settings for each patient remains challenging, and several unmet needs still exist in integrating STLs into routine clinical practice. In the present study, a wide power range for the PT was observed while achieving an adequate titration using STL, from which we aimed to determine personalized parameters that could maximize efficacy while minimizing side effects.

Two international guidelines on STL exist, one from the International Retinal Laser Society (LIGHT) [24] and the other from the Subthreshold Laser Ophthalmic Society (SOLS) [23], which propose different treatment approaches. The former advocates for using fixed parameters for all patients and discourages titration due to the possibility of unintended retinal damage [30,31]. Their publication includes a 577 nm micropulse laser (Iridex IQ577) with a recommended fixed power of 250 mW. Moreover, titration before treatment is more time-consuming than using fixed parameters and leads to extra burns with higher energy that, even placed away from the fovea, can induce potentially clinically significant retinal damage.

Conversely, SOLS advocates for personalized or titrated treatment, with the final power calculated as a fixed percentage (50%) of the energy required to achieve each patient’s PT, although other authors advocate for a 33% reduction in PT [25]. However, due to the moderate results of STL in CSCR shown in different studies, several authors do not consider the latter as a first-line therapy [13].

In the case of proposing a titration of 33% with fixed parameters at a power of 400 mW, this would involve assuming that most patients have a PT of 1200 mW. However, in our sample, 21.7% of the eyes had a PT of 1200 mW, while 58.7% had a PT below 1200 mW and 19.6% had one above. Only 21.7% of the eyes treated fell within the energy proposed by fixed regimens. Conversely, 58.7% needed less energy and would have been overtreated, while 19.6% needed more energy, risking undertreatment if fixed parameters were adopted.

An argument for titration is that it is possible to achieve personalized treatment while avoiding under- or overtreatment due to highly variable sensitivity to the energy received in STL. The clinical relevance of these energy differences remains uncertain. However, it is worth noting that in an animal study by Lavinsky et al. [2,3], at 30% of the PT energy, none of the laser spots showed any dead cells, but at 40% energy, damage was visible in 69% of the laser spots. In a real-world setting, our group very recently described the development of severe HRPE after STL in seven CSCR patients after using a 10% duty cycle [26].

Based on the results of the present study, the mean PT was 1102.7 ± 183.0 mW, with a wide range of energy used in the 92 eyes included (from 800 to 1600 mW). Only nine eyes (9.8%) with a PT of 800 mW used a treatment energy of around 250 mW, in accordance with the values proposed by the LIGHT. The remaining eyes in our sample (90.2%) would have been considered overtreated by their standards, with potential side effects such as retinal damage, hyperplasia of the RPE, choroidal neovascularization [32], retinal bleeding, fibrosis [33], and atrophy with irreversible vision loss. However, no visible retinal damage was observed in the OCT in our study, but the total resolution of STL in our group is far from 50%, with TR at 16.3%, PR at 24.5%, and GR at 40.8%.

In a multicenter study by the PACORES group [34], 92 eyes with CSCR were treated with two different yellow laser devices, using titration, and compared with half-dose PDT. The STL parameters employed were a duty cycle of 5%, a spot size that varied from 100 to 200 microns, a pulse duration of 200 ms, and power that ranged from 320 to 660 mW, 50% of the PT. They described the functional and anatomical results, but no data about PT were reported. Theoretically, the PT observed in their cohort ranged from 640 to 1320 mW, but there are no data about the PT distribution in their cohort. Our range for the PT is from 800 to 1600 mW, skewed towards higher energies. However, we calculated final energy using 33% instead of 50%, so the range of energy used in our treatments ranged from 265 mW to 528 mW. Using that scheme, PACORES reported that almost 50% of the eyes improved their final VA by 3 or more ETDRS letter lines. Our group has proven such improvement in 4.1% of the eyes studied. It is interesting that even with similar approaches to STL treatment, such disparities exist.

DSM can be challenging to differentiate from CSCR, as its clinical presentation is similar, with SRF in the subfoveal area. Some patients referred for STL were ultimately diagnosed with DSM. Various treatments have been employed for DSM, with several studies utilizing STL. Pirani et al. [10] employed a 577 nm STL in 11 eyes two weeks after half-fluence PDT. At 6 months, 45.4% achieved complete resolution, with all cases showing improvement in SRF. Rodrigues et al. [9] utilized a 532 nm STL, observing that, out of eight recruited eyes, they achieved a complete response in 87.5% of cases, with one eye showing partial resolution. Conversely, Battaglia Parodi et al. [8] utilized an 810 nm STL with titration. In their study, out of 12 recruited eyes, complete resolution of SRF was achieved in 1 eye, while the remaining cases demonstrated a partial resolution of SRF. As these results seemed highly efficacious, our group included DSM in the study sample to evaluate the possible differences between CSCR and DSM. However, the results of these articles contrast with those obtained in our sample, where 16.7% showed PR and 83.3% showed NR.

Our findings align with the PACORES study, which reported significant variability in STL titrations. However, our study provides a detailed analysis of the PT distribution, demonstrating that many patients require energy levels outside the range recommended by fixed-parameter guidelines. Furthermore, our results contrast with those of Pirani et al. and Rodrigues et al., who reported higher response rates to STL in DSM patients. These discrepancies could be attributed to differences in laser settings, patient selection, and follow-up duration, emphasizing the need for standardized yet adaptable STL protocols.

Regarding possible influencing factors, the PT did not appear to be influenced by sex, ocular laterality, CMT, or macular condition (CSCR and DSM), with only a weak correlation with SE and with age (*p* < 0.05). In this context, Takashina et al. [35] found that the time to complete pan-retinal photocoagulation, the CMT, the thickness of the upper quadrant, the nasal quadrant, and prior vitrectomy and cataract surgery were factors influencing the energy threshold required to achieve the PT in patients with diabetic macular edema. In a subgroup analysis of eyes with vitrectomy and cataract surgery, axial length (AL) was also significant.

The density of the RPE can vary depending on the examined region. While generally uniform, anatomical features and pathological conditions may cause variations. Within the vascular arcades, the increased structural complexity of the retina, due to a higher density of blood vessels and neural elements, can influence the perception of pigment density. Outside the arcades, the retinal layers are less complex, leading to a more homogeneous pigment distribution [36]. Therefore, titrating within the vascular arcades is crucial to ensure a pigment density comparable to that of the foveal zone. In addition, DSM in myopic eyes is closely linked to a posterior staphyloma, a localized scleral protrusion. While choroidal vascular alterations share similarities with CSC, the RPE’s density differs from emmetropic or hyperopic individuals, suggesting unique structural and functional adaptations in highly myopic eyes.

This is the first study that offers a database of the PT of using STL, both in CSCR and DSM, showing the distribution of energies and assessing the possible influence of demographic and ocular factors. We hope the data and values offered may help guide STL treatments to achieve better results. Based on the wide range of eyes falling outside the energy established by fixed-parameter regimens and the low percentage of complications seen using this strategy (avoiding a 10% duty cycle), the authors recommend titration from the first treatment to improve efficacy from the beginning, thus minimizing undertreatment, which would lead to persistent and refractory SRF with a subsequent loss in VA.

The implementation of personalized STL titration presents practical challenges, including the need for specialized training and precise imaging techniques to ensure accurate PT determination. Additionally, inter-device variability in terms of laser output further complicates the establishment of universal titration guidelines. Future research should focus on developing AI-assisted titration algorithms and real-time retinal response monitoring to enhance the accuracy and reproducibility of individualized STL protocols.

The present study has several limitations. First, there was a relatively small sample and an absence of different races. Unfortunately, we could not include other races as most of our patients were Caucasian, and we do not have a cohort from other ethnicities for comparison. Second, titration was challenging in patients with marked myopic choroidal changes despite efforts to target less atrophic-affected parenchymal areas, potentially affecting the interpretation of the optimal PT placement. Third, we did not have ALs to include in our analysis. In this regard, we could hypothesize that some relationship would exist, as the correlation between them is 0.8 [37]. Moreover, based on a pilot study, we decided to begin the titration at 800 mW, and we probably could have started at lower energies. However, as only nine eyes (9.8%) achieved the PT at 800 mW, and observing that the histogram of the distribution of the PT is similar to a normal distribution, with a bell shape (with only one peak, and it is symmetric around the mean), it was expected that less than 5% of the cases would titrate below 800 mW. In addition, it is essential to acknowledge that the observed results and titration power may vary depending on the specific laser device used and its particular characteristics. In this study, the Navilas 577s laser was employed, but other machines, such as the Iridex IQ577 or similar 577 nm lasers from different manufacturers, may have differing internal configurations and wiring, leading to variations in titration power. Therefore, the parameters and results presented here are specific to the Navilas device and may not be directly transferable to other laser systems. This variability in laser machines and differences in retinal pathologies make it challenging to establish a universally applicable protocol for subthreshold laser treatment. Finally, we would like to emphasize that the aim of the present study was to determine the photocoagulation threshold value rather than to assess the efficacy of the laser treatment. It is well known that a long-term follow-up is required to evaluate therapeutic responses, and multiple laser sessions are often necessary.

Future studies are warranted to analyze the heterogeneity in patients’ responses to STL and the factors influencing treatment outcomes to identify biomarkers that can predict which patients will benefit most. Standardized protocols often fail to account for individual differences, underscoring the need for tailored approaches based on specific retinal pathologies, disease stages, and patient characteristics. Our findings support the necessity of personalized titration and highlight the importance of using objective markers, such as choroidal thickness dynamics and autofluorescence changes, to refine STL adjustments. Exploring these multimodal imaging and functional biomarkers could help develop evidence-based STL guidelines that balance standardization with patient-specific adaptations, ultimately improving treatment efficacy and safety.

## 5. Conclusions

This study provides the first comprehensive database on STL photocoagulation threshold (PT) values in CSCR and DSM, offering valuable reference points for clinical practice. Our findings emphasize the need for individualized titration strategies, as the observed variability in the PT challenges the applicability of fixed-parameter approaches. Future research should focus on identifying biomarkers that predict STL responses, refining titration algorithms, and developing standardized yet flexible treatment protocols to optimize outcomes in diverse patient populations.

## Figures and Tables

**Table 1:** Multivariate regression analysis of the factors determining whether a greater or lesser power is needed to reach the photocoagulation threshold (PT).

**Dependent Variable: Power to Achieve PT (mW)**	**Unstandardized Beta**	***p***
Constant	927.365	<0.001
Age (years)	4.451	0.022 *
Gender (male/female)	−34.922	0.469
Refractive error (diopters)	−19.108	0.011 *
Macular condition (CSCR/DSM)	−13.568	0.836
Central macular thickness (µm)	0.002	0.992

* Statistically significant.

## Data Availability

The data presented in this study are available on request from the corresponding author.
